# YouTube as a Source of Medical and Epidemiological Information During COVID-19 Pandemic: A Cross-Sectional Study of Content Across Six Languages Around the Globe

**DOI:** 10.7759/cureus.8622

**Published:** 2020-06-15

**Authors:** Anirban Dutta, Nitya Beriwal, Linda M Van Breugel, Sonali Sachdeva, Bhupen Barman, Hiranya Saikia, Udeme-Abasi Nelson, Ahmed Mahdy, Subhankar Paul

**Affiliations:** 1 Medicine, Dr. NMB Baruah Nursing Home, Nalbari, IND; 2 Medicine, Lady Hardinge Medical College, New Delhi, IND; 3 Medicine, Leiden University Medical Centre, Leiden, NLD; 4 Medicine, North Eastern Indira Gandhi Regional Institute of Health and Medical Sciences (NEIGRIHMS), Shillong, IND; 5 Community Medicine/Biostatistics, Assam Medical College, Dibrugarh, IND; 6 Epidemiology and Public Health, University of Uyo Teaching Hospital, Uyo, NGA; 7 Internal Medicine, Al Andalus Polyclinics, Alexandria, EGY; 8 Medicine, Private Clinic, Hallidayganj, IND

**Keywords:** internet, youtube, patient education, quality of information, covid-19, sars-cov-2, coronavirus

## Abstract

Introduction

The current coronavirus disease 19 (COVID-19) outbreak has been declared to be a pandemic by the World Health Organization (WHO). It is evolving daily and has jeopardized life globally across social and economic fronts. One of the six key strategic objectives identified by the WHO to manage COVID-19 is to communicate critical information to all communities and prevent the spread of misinformation. We analyzed content on YouTube.com, a widely used web-based platform for medical and epidemiological information.

Methods

YouTube search results using two keywords were analyzed each in six languages - English, Arabic, Bengali, Dutch, Hindi, and Nigerian Pidgin on April 8, 2020. Forty videos in each of the six languages (i.e., a total of 240 videos) were included for analysis in the study. Two reviewers conducted independent analyses for each language. The inter-observer agreement was evaluated with the kappa coefficient (κ). Modified DISCERN index and Medical Information and Content Index (MICI) scores were used for the reliability of content presented in the videos and information quality assessment, respectively. Analysis of variance, Kruskal-Wallis, Mann-Whitney test, and chi-square tests were done appropriately for data analysis. A p-value of less than 0.05 was considered statistically significant. All calculations were performed using SPSS Statistics for Windows, Version 21.0 (IBM Corp, Armonk, NY).

Results

The videos cumulatively attracted 364,080,193 views. Altogether, 52.5% of videos were Informative, 23.75% were News Updates, and 8.33% were Personal Experiences. Ten percent of videos were found to present medically misleading information. Independent Users contributed 75% of the misleading content. The overall Mean DISCERN score, an index of content reliability, was 2.62/5. The overall Mean MICI Score was 5.68/25. Videos had better scores in the Transmission component of the MICI scale and scored low on the Screening/Testing component.

Conclusion

The reliability and quality of the content of most videos about COVID-19 and severe acute respiratory syndrome coronavirus 2 (SARS-CoV-2) were found to be unsatisfactory. Videos with misleading content were found across all six languages, and sometimes garnered a higher percentage of views than those from credible sources. The share of videos contributed by Government and Health Agencies was low. Medical institutions and health agencies should produce content on widely used platforms like YouTube for quality medical and epidemiological information dissemination.

## Introduction

The cluster of patients afflicted with the novel coronavirus was initially reported on December 31, 2019, to the World Health Organization (WHO) China Country Office. These cases were from the city of Wuhan in the Hubei province of China [1]. A novel strain of coronavirus isolated on January 7, 2020, was implicated as the probable infectious agent [1]. This cluster sequentially spread beyond the boundaries of China to become a global healthcare emergency. On February 11, 2020, the virus was renamed as severe acute respiratory syndrome coronavirus 2 (SARS-CoV-2) by the International Committee on Taxonomy of Viruses. The disease caused by it was labeled as coronavirus disease (COVID-19) by the WHO on the same day [2]. Exactly one month later, COVID-19 was declared to be a pandemic by the WHO [3]. Centers for Disease Control and Prevention (CDC) highlights that the virus mainly spreads from person to person via the mode of respiratory droplets. This can occur upon being within a circumference of two meters or six feet of a COVID-19 patient for a prolonged period or by having direct contact with infectious respiratory secretions [4].

One of the six key strategic objectives identified by the WHO to manage COVID-19 is to communicate critical information to all communities and prevent the spread of misinformation [5]. The internet is the most accessible form of information available to all at the click of a button and is increasingly being used by the masses to procure and understand health-related information. It has been shown that web-based interventions are associated with improved outcomes to achieve specified knowledge and behavior change as compared to those interventions which are not web-based. These results were projected to several parameters, one of the key parameters being increased participation in healthcare and medical knowledge [6].

Reports from Alexa, an Amazon global company, reports YouTube as the second most visited web portal after Google.com in its top 50 global websites as of March 29, 2020 [7]. Some studies have analyzed the impact of YouTube in previous pandemics. A study by Pandey et al. reported that YouTube served as a source of substantial useful information during the H1N1 influenza pandemic in 2009. A source-based preference and increased viewership were also noted for useful videos, unlike the misleading ones [8]. On the contrary, in a study by Bora et al., which analyzed YouTube video content during the Zika virus 2015 pandemic, it was shown that a considerable quantity of videos on YouTube was misleading. Furthermore, such videos had higher viewership compared to informative videos [9]. These studies with contradictory findings highlight that YouTube can be a means to disseminate vital critical information. However, it may also lead to the spread of misinformation that needs to be analyzed and controlled in emergencies of worldwide public concern.

A Google Trends search on March 29, 2020, at 10 pm EST for most searched terms on YouTube with the filters ‘worldwide’ and ‘past 90 days’ yielded ‘coronavirus’ and ‘virus’ as the top two results, respectively. There was a 1600% rise in the search frequency of the term ‘virus’ while the term ‘coronavirus’ was denoted as ‘breakthrough.’ According to Google, “results marked ‘breakthrough’ had a tremendous increase, probably because these topics are new and had few (if any) prior searches” [10]. We are aware of at least one similar study having been carried out during the COVID-19 pandemic by Khatri et al. [11]. However, since the completion of said study, the outbreak has scaled up to affect almost all countries in the world and has been declared a ‘pandemic.’ The need for an elaborate wider view study was thus felt, and the team of researchers from different parts of the world (India, Egypt, Netherlands, and Nigeria) worked with an objective of ‘assessing the quality and reliability of YouTube videos on medical and epidemiological information during the COVID-19 pandemic.’ In alignment with the key strategic objective highlighted by the WHO, the aim of the study was to analyze the usefulness of YouTube as a web-based platform for medical and epidemiological information [5]. We also sought to analyze its role in the spread of misinformation, if any.

## Materials and methods

Videos were searched on YouTube on April 8, 2020, using two keywords, each in six languages: English, Arabic, Bengali, Dutch, Hindi, and Nigerian Pidgin [12].

The following keywords were used: ‘Coronavirus’ and ‘Corona Virus’ (English), كوفيد and كرونا (Arabic) translated in English as ‘COVID’ and ‘Corona’ respectively; করোনাভাইরাস and করোনা ভাইরাস (Bengali) translated in English as ‘Corona Virus’ and ‘Coronavirus’ respectively; ‘Coronavirus’ and ‘Corona Virus’ (Dutch), कोरोना वायरस and कोविड 19 (Hindi) translated into English as ‘Corona Virus’ and ‘COVID 19’; ‘Coronavirus’ and ‘Covid-19’ (Nigerian Pidgin). Two search terms related to the COVID-19 pandemic that yielded the maximum number of results were selected as ‘keywords’ for each language.

Videos were viewed after clearing the cache of the respective browsers and using a new YouTube account to minimize results biased by cookies, personal preferences, and browser history. Around 90% of the users of internet search engines view results within the first three pages of search results [9]. However, YouTube no longer uses pages to demonstrate results but does so in the form of a continuous list. Therefore, with the consideration of getting an adequate number of videos for strong statistical analysis, the top 120 search results yielded by the keywords were screened. The list of video results thus found was saved to avoid discrepancies later as the YouTube search algorithm would likely yield different results with further development of the pandemic [13]. Uniform Resource Locators of all the video samples included in the study were saved for purposes of data archiving and future referencing. As is common for any ongoing pandemic, several videos in the results addressed issues of non-medical nature (e.g., socioeconomic and political aspects).

Thus, only the initial forty videos fulfilling specified inclusion and exclusion criteria were considered for analysis. Results on searching specific defined keywords in the respective languages were analyzed. Content of selected videos fell into one or more of the following categories: epidemiology, clinical features, prevention, management strategies, experience with the disease, or latest news updates on COVID-19.

We excluded videos whose content was not medically related to the COVID-19 disease (e.g., videos on political aspects, and disease impact on the economy). Partially or fully duplicated videos were omitted as well.

Important video characteristics including the title of the video, uploader/channel name, number of views, upload date, views/day, duration of the video, number of likes and dislikes, like-dislike ratio, and number of comments were documented. Any scientifically inaccurate statement made in the video was also transcribed (and translated into English wherever applicable).

Evaluation of the sampled videos

Each video was independently reviewed by two authors or volunteers of a medical background, with both being proficient in the language used in the videos. The videos were graded on their reliability and quality of content.

The reliability of the information provided by the videos was graded on a scale adapted from the modified DISCERN tool used in previous studies on similar subjects [9,11,14]. The tool consists of five questions that are answered with a “yes” or “no” response and scored as 1 (one) point for an affirmative answer and 0 (zero) points for a negative answer. Each video was therefore graded from zero to five, with zero indicating low reliability and five indicating high reliability. Appendix Table 8 shows the questions adapted from DISCERN tool used for the evaluation of the reliability of the videos

The content of the videos was assessed using the Medical Information and Content Index (MICI) scale, which was devised by Nagpal et al. for a similar study on the Ebola Hemorrhagic Fever epidemic [13]. The MICI scale uses a five-point Likert scale from one (indicating poor quality) to five (indicating high quality) to assess five components of information included in the videos: prevalence, transmission, clinical symptoms, screening/testing, and treatment/outcomes of the infection.

These five components were graded using criteria adapted from a similar study done by Khatri et al. [11]. Publications and guidelines from the CDC and WHO were used as reference material for developing the criteria for the five components of the MICI scale (Appendix Table 9).

After a thorough review of existing literature, the videos were classified into four non-overlapping groups: Informative-content that conveys medically correct information about one or more aspects of the disease including epidemiology, prevention, clinical features, screening and testing and treatment to its viewers; Misleading-content that is scientifically inaccurate or makes ambiguous claims that are not evidence-based; Personal Experiences-content primarily based upon the individual’s own experience or that of family members/relatives/friends/neighbors suffering from COVID-19; and News Updates-content focused on giving the latest updates about the disease burden and mortality only without addressing symptomatic, preventive or management aspects of COVID-19.

Videos were also classified according to their sources into independent users, government or health agencies, news agencies, hospitals or academic institutions, and medical advertisement/for-profit companies.

Data analysis

The data were presented as counts, percentages, mean ± standard deviation (SD), and median (interquartile range) depending on the nature of the data. Comparisons of the mean values among the various groups were made by one-way analysis of variance. In contrast, the distribution of the median across the groups was tested by Kruskal-Wallis test, and between the groups was tested by the Mann-Whitney test. The significance of the differences among the proportions was tested by the chi-square test. A P value of less than 0.05 was considered statistically significant. Kappa-coefficient (κ) was used to see the degree of agreement between the two researchers. All calculations were performed using SPSS Statistics for Windows, Version 21.0 (IBM Corp, Armonk, NY).

## Results

Forty videos in each of the six languages (i.e., a total of 240 videos) were included for analysis in the study. This was done after screening 120 top results yielded by the keywords in each of the languages. The kappa coefficient of agreement between researchers was found to be significant in all six languages (P < .01) (Table 1).

**Table 1:** Kappa coefficient (κ) of YouTube videos across all six languages studied

Language	Kappa (κ)	P value
Arabic	0.85	P < .01
Bengali	0.76	P < .01
Dutch	0.70	P < .01
English	1.00	P < .01
Hindi	0.82	P < .01
Nigerian Pidgin	0.88	P < .01
Overall	0.88	P < .01

The cumulative number of views was 364,080,193. Videos in Hindi accumulated the highest number of views (210,956,181), while those in Nigerian Pidgin had the fewest (5,619).

The median number of likes per video was 445 (range, 0 to 84,800), while the median number of comments per video was 65.5 (range, 0 to 46,300). The median duration of the videos was 4 minutes, 40 seconds (range, 30 seconds to 79 minutes, 2 seconds). The median number of views per day was 3,350 (range 0 to 1,760,891) (Table 2). 

**Table 2:** Video characteristics and viewer interaction metrics of YouTube videos analyzed across all six languages studies IQR: interquartile range.

	Total number of views	Median number of views per video (IQR)	Median duration of videos in mm:ss (range)	Median number of comments per video (IQR)	Highest number of views in one video	Median likes:dislikes ratio (IQR)
English (n = 40)	110,255,134	894,306 (427,222 - 3,216,236)	6:41 (01:21 - 50:38)	1907.5 (463.2 - 5230.5)	22,782,222	15 (10.9 - 23.1)
Arabic (n = 40)	11,368,543	14,110.5 (2,901.8 - 104,580)	6:59 (01:17 - 29:05)	51 (8.8-79)	4,305,344	26 (6.8 - 29.2)
Bengali (n = 40)	20,220,493	112,004 (20,704.8 - 395,911)	3:26 (01:15 - 21:25)	54.5 (20.6-168)	8,739,400	13.89 (10.7 - 26.7)
Dutch (n = 40)	11,116,426	143,289 (30,541.8 - 406,518)	3:32 (01:08 - 79:02)	564 (65.5-933)	1,815,420	14.34 (6.7 - 28.8)
Hindi (n = 40)	210,956,181	2,717,575 (159 - 7,813,718)	2:22 (0:30 - 27:16)	861 (0-4041)	14,204,108	4.14 (0 - 8.7)
Nigerian Pidgin (n = 40)	5,619	14.5 (3.5 - 65)	3:05 (0:56 - 18:58)	0 (0-1)	2,700	0 (0 - 0)

Altogether, 139 (52.5%) videos were classified as informative, 57 (23.75%) as news updates, and 20 (8.33%) as personal experiences. Twenty-four videos (10%) were flagged as misleading (Table 3). Eighteen (75%) of these were from independent users, while news agencies uploaded six (25%). No misleading content was uploaded by government/national/international health agencies, nor by hospitals and academic institutions.

**Table 3:** Classification of videos based on content across all six languages studied N: number of videos; n%: percentage of video category with respect to total videos of a particular language; VN: number of views across video category in each particular language; v%: percentage of video category with respect to total videos of a particular language.

	Informative		Misleading		News update		Personal experience	
	N (n%)	VN (v%)	N (n%)	VN (v%)	N (n%)	VN (v%)	N (n%)	VN (v%)
English	23 (57.5)	79,116,941 (71.76)	2 (5)	4,408,473 (3.99)	8 (20)	11,019,001 (9.99	7 (17.5)	15,827,454 (14.26)
Arabic	17 (45)	5,308,585 (46.7)	5 (12.5)	48,888 (0.43)	13 (27.5)	5,858,659 (51.51)	5 (15)	157,191 (1.36)
Bengali	34 (85)	18,278,492 (90.39)	3 (7.5)	1,634,592 (8.1)	1 (2.5)	26,789 (0.1)	2 (5)	280,620 (1.41)
Dutch	33 (82.5)	8,932,968 (80.36)	1 (2.5)	69,805 (0.63)	3 (7.5)	760,304 (6.9)	3 (7.5)	1,353,349 (12.11)
Hindi	6 (15)	19,281,395 (9.14)	6 (15)	87,546,815 (41.50)	28 (70)	104,001,397 (49.34)	0 (0)	0 (0.02)
Nigerian Pidgin	26 (65)	4,557 (81.1)	7 (17.5)	404 (7.3)	4 (10)	601 (10.7)	3 (7.5)	51 (0.9)

The overall mean DISCERN score was 2.62 ± 1.32. The highest number of desirable (e.g., yes) responses were for item number three of the DISCERN tool (195, 81.25%) while the fewest were for item number four (51, 21.25%). The cumulative score of 0, which indicates the lowest reliability, was met by 22 videos (9.16%). The highest cumulative score of five, implying high reliability, was fulfilled by 33 videos (13.75%). Videos in Dutch had the highest mean DISCERN Score (3.35 ± 1.07) among the six languages, while those in Hindi had the lowest (2.08 ± 0.94). Informative videos had a significantly higher total mean DISCERN score compared to other categories. (P < .01) (Table 4).

**Table 4:** Mean modified DISCERN scoring of YouTube videos analyzed across all six languages studied SD: standard deviation.

	Mean DISCERN score on item 1 (SD)	Mean DISCERN score on item 2 (SD)	Mean DISCERN score on item 3 (SD)	Mean DISCERN score on item 4 (SD)	Mean DISCERN score on item 5 (SD)	Mean Total DISCERN score (SD)
English (n = 40)	0.85 (0.36)	0.55 (0.50)	0.88 (0.33)	0.22 (0.42)	0.48 (0.50)	2.98 (1.44)
Arabic (n = 40)	0.8 (0.40)	0.58 (0.50)	0.55 (0.50)	0.38 (0.49)	0.5 (0.50)	2.75 (1.54)
Bengali (n = 40)	0.5 (0.51)	0.55 (0.50)	0.92 (0.27)	0.12 (0.33)	0.22 (0.42)	2.32 (1.20)
Dutch (n = 40)	0.9 (0.30)	0.6 (0.49)	0.92 (0.27)	0.18 (0.38)	0.78 (0.42)	3.35 (1.07)
Hindi (n = 40)	0.9 (0.30)	0.2 (0.40)	0.92 (0.27)	0 (0)	0.15 (0.36)	2.08 (0.94)
Nigerian Pidgin (n = 40)	0.78 (0.42)	0.25 (0.44)	0.68 (0.47)	0.38 (0.49)	0.22 (0.42)	2.3 (1.60)
Mean scores of all languages	0.78 (0.41)	0.45 (0.50)	0.81 (0.39)	0.21 (0.41)	0.39 (0.49)	2.62 (1.32)

The mean MICI score was 5.68 ± 4.22. None of the videos achieved the highest possible score of 25, while 27 videos (11.25%) scored the lowest possible score of 0. The highest MICI score of 18 was attained by five (1.1%) videos. Overall, videos had better scores in the transmission component (mean score 1.90 ± 1.45) of the MICI scale and fared poorest on the screening/testing component (mean score 0.46 ± 0.92) (Table 5).

**Table 5:** Mean medical information and content index scoring of YouTube videos analyzed across all six languages studied Note: Outcomes include the current non-availability of a vaccine. MICI: medical information and content index; SD: standard deviation.

	Mean MICI score on prevalence (SD)	Mean MICI score on transmission (SD)	Mean MICI score on clinical symptoms (SD)	Mean MICI score on screening/testing (SD)	Mean MICI score on treatment/outcome (SD)	Mean total MICI score (SD)
English (n = 40)	1.2 (1.42)	2.3 (1.60)	2 (1.50)	0.35 (0.77)	1.28 (1.37)	7.12 (4.71)
Arabic (n = 40)	1.48 (1.34)	1.8 (1.36)	1.32 (1.22)	0.92 (1.16)	1.55 (1.28)	7.05 (4.64)
Bengali (n = 40)	0.6 (1.03)	1.68 (1.58)	0.92 (1.12)	0.3 (0.88)	0.65 (1.08)	4.15 (3.34)
Dutch (n = 40)	1.1 (1.72)	2.08 (1.40)	1.58 (1.48)	0.62 (1.03)	1.48 (1.20)	6.88 (4.05)
Hindi (n = 40)	1.1 (0.96)	1.35 (1.39)	0.50 (0.96)	0.55 (0.98)	0.42 (1.13)	3.90(3.78)
Nigerian Pidgin (n = 40)	0.52 (0.99)	2.22 (1.20)	1.62 (1.39)	0 (0)	0.68 (0.79)	5.02 (3.47)
Mean scores of all languages	1.00 (1.99)	1.90 (1.45)	1.32 (1.37)	0.46 (0.92)	1.01 (1.23)	5.68 (4.22)

One hundred twenty-seven (52.91%) videos addressed the prevalence of the COVID-19. One hundred eighty-five (77.0%) videos addressed one or more aspects of the transmission of SARS-CoV-2, including basic precautionary measures like handwashing and social distancing.

Ninety-eight (40.83%) videos mentioned the signs and symptoms of COVID-19. Only five videos (2.08%) addressed all the following components-common symptoms, less common symptoms, emergency signs of COVID-19 that require medical attention urgently, and that some people may get infected but do not develop the disease. One hundred twenty-seven (52.91%) videos mentioned treatment and outcomes.

Misleading videos had significantly lower total mean MICI scores compared to informative videos, news updates, or personal experience videos (P < .01). They also had significantly lower coverage and lower MICI scores of the prevalence, transmission, clinical symptoms, and treatment/outcome components of the MICI scale (P < .05). There was no significant difference in MICI scores among the four groups regarding the screening/testing component (P = .550) (Table 6).

**Table 6:** Assessment of healthcare based characteristics of all videos based on content and quality based categorization MICI: medical information and content index; SD: standard deviation.

	Informative (n = 139)	Misleading (n = 24)	News update (n = 57)	Personal experience (n = 20)	P value
Mean MICI score on prevalence (SD)	0.89 (1.12)	0.83 (1.09)	1.44 (1.31)	0.70 (1.30)	P < .05
Mean MICI score on transmission (SD)	2.28 (1.43)	1.29 (1.40)	1.46 (1.31)	1.30 (1.34)	P < .01
Mean MICI score on clinical symptoms (SD)	1.68 (1.39)	0.71 (1.23)	0.79 (1.19)	1.10 (1.12)	P < .01
Mean MICI score on screening/testing (SD)	0.47 (0.98)	0.21 (0.51)	0.51 (0.95)	0.55 (0.89)	P = .550
Mean MICI Score on treatment/outcome (SD)	1.20 (1.29)	0.58 (1.06)	0.81 (1.16)	0.75 (0.91)	P < .05
Mean total MICI score (SD)	6.51 (4.14)	3.58 (3.80)	4.96 (4.18)	4.40 (4.12)	P < .01
Mean total DISCERN score (SD)	3.01 (1.22)	1.17 (1.52)	2.56 (1.15)	1.90 (1.52)	P < .01
Coverage of various aspects of MICI:					
Prevalence (%)	69 (49.6)	11 (45.8)	40 (70.2)	7 (35.0)	P < .05
Transmission (%)	118 (84.9)	16 (66.7)	38 (66.7)	13 (65.0)	P < .05
Clinical symptoms (%)	99 (71.2)	8 (33.3)	23 (40.4)	12 (60.0)	P < .01
Screening/testing (%)	33 (23.7)	4 (16.7)	19 (33.3)	7 (35.0)	P = .277
Treatment/outcomes of the infection (%)	87 (62.6)	7 (29.2)	23 (40.4)	10 (50.0)	P < .01

News agencies were the single largest contributors of content (142, 59.17%), followed by independent users (64, 26.67%). Government and national/international health agencies from around the world contributed only seven videos (2.92%). Hospital and academic institutes uploaded 13 videos (5.42%), and medical advertisement companies or other for-profit companies accounted for 14 videos (5.83%) (Table 7).

**Table 7:** MICI and DISCERN scores according to video source MICI: medical information and content index; SD: standard deviation.

	Independent user (n = 64)	Government or health agencies (n = 7)	News agencies (n = 142)	Hospitals or academic institutions (n = 13)	Medical advertisement/for-profit companies (n = 14)	P value
Mean MICI score on prevalence (SD)	0.73 (1.07)	0.43 (0.78)	1.20 (1.26)	0.46 (0.52)	1.00 (1.41)	P < .05
Mean MICI score on transmission (SD)	2.13 (1.41)	2.00 (1.92)	1.87 (1.44)	1.38 (1.66)	1.71 (1.38)	P = .480
Mean MICI score on clinical symptoms (SD)	1.72 (1.50)	1.14 (1.57)	1.20 (1.27)	1.00 (1.47)	1.21 (1.48)	P = .114
Mean MICI score on screening/testing (SD)	0.34 (0.78)	1.86 (1.68)	0.43 (0.88)	0.77 (1.24)	0.29 (0.73)	P < .01
Mean MICI score on treatment/outcome (SD)	0.94 (1.17)	1.71 (1.50)	1.05 (1.25)	0.77 (1.17)	0.79 (1.19)	P = .459
Mean total MICI score (SD)	5.84 (3.96)	7.14 (6.57)	5.71 (4.18)	4.38 (4.25)	5.00 (4.66)	P = .642
Mean total DISCERN score	2.31 (1.61)	4.29 (0.76)	2.77 (1.22)	2.54 (1.33)	1.93 (1.33)	P < .01

Most social media websites, including YouTube, provide primarily user-generated content. YouTube is a truly global platform, where videos are uploaded in languages from across the world daily. The six languages analyzed in the study, namely English (335 million speakers), Hindi (258 million speakers), Bengali (189 million speakers), Arabic (223 million speakers), Nigerian Pidgin (75 million speakers), and Dutch (24 million speakers), are spoken by approximately 1.104 billion people. One or more of these languages are spoken by native populations in all six permanently inhabited continents of the world [15-17]. Considering new information about the disease emerging on a daily to weekly basis, scientific accuracy can prove challenging to gauge. For this study, the content of websites of the CDC and the WHO was considered as standard and scientifically up-to-date.

## Discussion

In our study, we found an unprecedented number of views on the videos. The highest number of views in our study was in Hindi (210,956,181), which had almost twice as many views as English (110,255,134). Furthermore, the total number of views of English videos in our study was much higher than that of English videos in similar studies carried out during previous disease outbreaks and in the earlier stage of the present pandemic [9,11,13,18,19]. Similarly, we recorded higher viewer interaction metrics (total number of likes, the total number of comments). This shows the tremendous surge in usage of YouTube during the COVID-19 pandemic and scope for future studies in languages other than English. There was no significant difference in the like-dislike ratio between the Informative and Misleading videos (P = .364), one reason for which could be the inability of the viewers to identify the content as misleading.

The overall mean modified DISCERN Score of 2.62 ±1.32 indicates that the YouTube content analyzed suggested poor reliability, similar to Khatri et al. [11]. It is encouraging that 195 (81.25%) of the videos presented the information in a balanced and unbiased fashion. However, only 51 (21.25%) of them mentioned additional sources of information for the viewers (e.g., link to CDC or WHO websites), and only 109 (45.42%) used reliable sources of information (cited publications or the presenter was a medical or public health expert). The fact that 9.16% of the videos scored zero on the scale is alarming.

The mean MICI Score was low in all the six languages, implying a dearth of videos with good content. Videos in English and Arabic had the two highest mean ± SD MICI scores of 7.12 ± 4.71 and 7.05 ± 4.64, respectively, while those in Hindi fared the poorest with a mean score of 3.90 ± 3.78. The overall mean ± SD MICI score of 5.68 ± 4.22 was lower than reported in previous studies [11,13]. The screening and testing component had a low mean score of 0.46 out of five on the MICI scale. One hundred seventy-seven (73.75%) videos made no mention of any aspects of screening or diagnosis of COVID-19 at all. Although this was found to be very high, even higher numbers have been reported by Khatri et al. (90%) for the Mandarin language for the same pandemic [11]. This is very worrisome as WHO has highlighted the need for accurate diagnosis and effective isolation of patients to ‘slow the transmission of the disease and protect health systems’ [20].

News channels or agencies (e.g., British Broadcasting Corporation [BBC], The New York Times, and Cable News Network [CNN]) accounted for most of the uploaded videos (142, 59.17%). Videos from government and national/international health agencies had a higher MICI score (P = .642) and significantly higher DISCERN score (P < .01) than videos uploaded from other sources. However, their share of the videos was low (n=7, 2.91%). This is in congruence with previous studies [9,11,18,19]. Moreover, the videos from these agencies had a modest number of views when compared to some of the videos that bore misleading content. This could be because of the fewer subscribers to the YouTube channels of these agencies compared to some popular channels of independent users or news agencies. YouTube can investigate boosting the reach of videos from such credible sources to improve the purview of these videos.

We came across scientifically inaccurate content in all six languages (Appendix Table 10). The share of videos containing misleading information (n=24, 10%) was found to be similar to that reported by Khatri et al. (8%) but lower than studies analyzing YouTube content for the Zika virus pandemic of 2015 (23.8%), West Nile Virus outbreak of 2012 (20.76%), H1N1 pandemic of 2010 (23%), Sjogren’s syndrome (16.7%), pelvic organ prolapse (18%), kidney stones (18.1%), rheumatoid arthritis (30.4%), hypertension (33%), and atopic eczema (48%) [8,9,11,14,19,21-24]. Viewership of misleading content varied widely among the languages. The misleading videos in Arabic (n=5) and Dutch (n=1) garnered only 0.43% and 0.63% of the viewership, while such videos made up 41.50% of the viewership in Hindi (n=6). Hindi is a major language in India, which contributes a large section of YouTube viewership, and such a high percentage of misleading content should raise serious concerns [25].

Incorrect information about almost all aspects of the disease was found. Inaccuracies ranged from lapses (e.g., a video stated social distancing of six meters instead of six feet) to brazen sensationalizing conjectures (e.g., video surmises that the virus is related to the development of 5G wireless communication technology). We came across one video that insinuated that the viewer needed to be prepared for shortages “to protect their families” and promoted commercial products for emergency preparedness. Subsequent viewing of these videos two weeks later showed that most of these were still on YouTube, and only one video had been taken down for violating YouTube’s terms of service. YouTube has acknowledged its crucial role in the dissemination of information in the setting of a pandemic. They have taken steps to moderate content about SARS-CoV-2 and COVID-19 by making certain unreviewed content not available via search, on the homepage, or in recommendations [26,27].

The lower percentages of misleading videos do not paint the complete picture. Fully 79% of the viewers browse through more pages if they do not find their desired content on the first page [9]. Because the videos in all six languages frequently failed to address all or most aspects of the disease, the viewers would be likely to browse through more videos, exposing them to content from more sources. This may confuse the viewers by introducing them to multiple or misleading videos. Curbing of misinformation completely on online platforms can prove difficult even with today’s technology, especially in videos that only promote anecdotal narratives and do not make overt false claims. The tone of the presentation in such videos may be the chief means of propagation of misinformation. Most studies analyzing the content of YouTube as a tool for medical information have confined their research to English alone. However, our study shows that misleading content is generated in languages and regions around the world; in some cases, these non-English languages may garner a large proportion of the viewership. This bears even more significance in the setting of pandemics as information must be presented in a correct as well as timely fashion, leaving little margins for error or time.

There is a need for more videos from credible sources that address all or most aspects of the disease. Academic institutions and hospitals should recognize the importance of social media platforms like YouTube as tools for dissemination of information and actively seek to produce content in more languages seeking help from multidisciplinary experts readily available in their institutes. Credible content from academic institutions or national/international health agencies can serve as comprehensive sources of information, and they will be deemed trustworthy by the viewers if recommended by their doctors. Doctors can recommend such standard videos for patient reference if they are promoted among the medical fraternity in the initial stages of disease outbreaks by said agencies. By not having to look up multiple sources for information, the viewers would also be less likely to come across videos containing misinformation.

There were limitations to our study. For the sake of uniformity in all the languages, we chose only the two keywords that yielded the maximum number of results in a language. For example, in English, the search terms “coronavirus” and “corona virus” yielded the maximum number of results on YouTube and were thus selected. However, there were several other terms (e.g., COVID-19, Corona) pertinent to the disease that also yielded high numbers of results.

The cross-sectional design of the study restricted us from exploring the development of the videos in the user interaction aspects. It would have been insightful to note the number of likes, comments, and views with the evolution of the pandemic. Furthermore, YouTube takes down videos that violate their Community Guidelines after reviewing content reported by viewers [28]. Our study was conducted a few months into the development of this disease. Based on that, there is a possibility of an even higher number of misleading videos uploaded in the past that had been taken down before our analysis.

The six languages in this study are the ones in which the authors are proficient. Some languages have notably lower numbers of speakers than others. Nevertheless, we achieved linguistic representation from all the six major continents in the study.

## Conclusions

YouTube has experienced unprecedented viewership during the COVID-19 pandemic of 2019-20. The reliability and quality of the content of most videos about COVID-19 and SARS-CoV-2 were found to be unsatisfactory. Videos garnered low scores on the reliability scale and frequently failed to address key medical aspects of the disease. The total percentage of misleading videos was lower than in previous epidemics, but scientifically inaccurate content was found across all six languages studied. In one language, they accounted for almost half of the viewership. The share of videos contributed by government and health agencies was low. Our study shows the need for moderation of YouTube content and generation of videos with comprehensive medico-epidemiological information in languages other than English. The medical fraternity should take note of the tremendous reach of YouTube and utilize it as a tool for the circulation of key information among the masses from the incipient stages of a disease outbreak. Medical institutions and health agencies should seek to produce more informative content keeping in mind a global as well as local audience. They should also make their videos available on commonly used platforms like YouTube for widespread access to the general population.

## Appendices

**Table 8:** Questions adapted from DISCERN tool for the evaluation of the reliability of the videos 1 point is awarded for 'yes' response and 0 points awarded for 'no' response Adapted from [9,11,14]

Item	Questions
1	Are the aims clear and achieved?
2	Are reliable sources of information used? (i.e. publication cited, the speaker is a specialist e.g. doctor, nurse, public health expert, infectious diseases expert, virologist, microbiologist)
3	Is the information presented balanced and unbiased?
4	Are additional sources of information listed for patient reference?
5	Are areas of uncertainty mentioned?

**Table 9:** MICI scale to assess the quality of the content of videos Each item is awarded 1 point if mentioned in the video. A maximum score of 5 in each component. MICI: medical information and content index; COVID-19: coronavirus disease. Adapted from [11,13].

Component	Description of scoring
Prevalence	Number of globally/locally confirmed cases reported
	Number of globally/locally suspected cases reported
	Number of global/local deaths reported
	Mentions about populations at high risk
	Number/proportion of patients who are severely ill
Transmission	Location of origin of virus
	Human to Human Transmission (including spread via droplets)
	Mentions about spread from contact with contaminated surfaces
	Mentions about basic precautionary measures (wearing mask handwashing and social distancing)
	Mentions about Incubation period
Signs and Symptoms	Common symptoms: fever, tiredness, dry cough
	Other symptoms: shortness of breath, aches and pains, sore throat
	Less common symptoms: diarrhea, nausea or a runny nose
	Emergency warning signs for COVID-19 that require medical attention immediately (like Trouble breathing, Persistent pain or pressure in the chest, new confusion or inability to arouse, bluish lips or face)
	Mentions that Some people become infected but do not develop any symptoms and don't feel unwell
Screening/ Testing	Mentions there is a test available
	Mentions the test uses respiratory secretion to test
	Uses PCR to check identify SARS-CoV-2
	Shows how the test is done
	Mentions criteria for testing/screening
Treatment/ Outcome	Mentions that mild symptoms can be self-resolving
	Mentions that some patients become more ill (mentions Hospitalization, ICU admission) and die
	Mentions that treatment is supportive
	Mentions that vaccines are in the making – none currently available
	Mentions about rational use of medical masks

**Table 10:** Transcript of misleading statements found in videos across all six languages

Misleading Statements
Gargle with warm water and salt to protect yourself from the virus
Do not trust hand sanitizers
Chloroquine treats the disease
The virus can be treated by antibiotics
The virus is a result of biological war
Dead bodies can spread the virus
The virus is related to the development of 5G technology
The virus is not very contagious
Family members often do not get sick
The virus cannot survive outside the body, so cannot spread via objects
You can visit busy places without having to worry about getting sick
Lemon, papaya, broccoli, Indian gooseberry, spinach help prevent getting the infection
Coronavirus does not survive above 21 degrees Celsius for more than five days so our country is safe
The virus was created in a lab
Wash your hands for twenty minutes
Must maintain social distancing of 6 meters
The virus spreads like a ‘bushfire’ during the dry season
When the disease becomes an emergency, the patient will develop fever
Deep breathing and chanting of “Om” identifies patients
Antidote found in Thailand
Spread from Bat attack on a scientist
Sunlight cures all diseases
The disease is not different from regular flu
Hot water kills the virus
Pangolin transmits the disease
